# The form of *good*

**DOI:** 10.1007/s11098-025-02474-x

**Published:** 2026-01-09

**Authors:** Poppy Mankowitz

**Affiliations:** University of Bristol, Bristol, UK

**Keywords:** ‘Good’, Value, Syntactic arguments, ‘Tough’-adjectives, Context-dependence

## Abstract

Some philosophers hold that sentences with the word *good* have a uniform form. On this view, many of the apparent syntactic and semantic differences between (say) *That is a good knife*, *Xavier is good with children* and *It is good to have pets* are illusory. A difficulty in evaluating uniformity theses is that they are often not formulated in a linguistically precise way. I provide an interpretation where uniformity theses treat *good* as taking the same arguments at some syntactic or semantic level. I then defend the view that the motivation for uniformity theses is weak, and I develop a strategy for opposing them. One version of this strategy is deployed, drawing on under-appreciated data about *tough*-adjectives. I argue that there is better motivation for alternative analyses of *good*, namely ‘non-uniformity contextualism’ and ‘relativism’. The resulting picture is one where the apparent syntactic and semantic differences between *good*-constructions are genuine.

## Introduction

The word *good* is important in philosophy. After all, goodness and value are of perennial interest, and the words that we use to talk about them might illuminate aspects of their nature. A number of philosophers have sought an analysis where all sentences with the word *good* have a uniform form. This analysis is often paired with certain views about goodness, such as that only one type of thing—say, states of affairs—can be bearers of value, or that value is always relativised to features of the context. The current paper argues that an investigation of the syntax and semantics of *good* opposes uniformity theses and motivates alternative analyses.

The constructions in which *good* occurs appear diverse. Here is a sample:

1. a. Liquorice is good to eat.
  b. Xavier is good to Ylva.
  c. Vitamin C is good for children.
  d. That knife is good for cutting chives.
  e. Xavier is good with children.
  f. Ylva is good at playing poker.
  g. That rock is good as a paperweight.
  h. That is a good knife.
  i. It is good that Xavier has pets.
  j. It is good to have pets.
  k. It is good for Xavier to have pets.
  l. Pleasure is good.
  m. God is good.

Despite this apparent diversity, some philosophers claim that there is something uniform about the form of these constructions. For instance, it has been suggested that the meanings of *good* sentences always involve the contribution of a noun (Geach, 1956) or always involve a relation between three propositions (Finlay, 2014). The resulting picture is one where the semantics, and possibly the syntax, of *good*-constructions show fundamental uniformity. Many of the apparent syntactic and semantic differences between (1a)–(1m) are illusory.

Similar claims have been made about a uniform form for sentences with other philosophically important words, such as *ought* (Finlay & Snedegar, 2014), *true* (Prior, 1967), intentional attitude verbs like *believe* (Quine, 1956) and attitude adjectives like *afraid* (Nebel, 2019). The fact that these sorts of claims occur across a variety of debates makes it all the more important to subject them to scrutiny. Claims about a uniform form are hard to evaluate, because they are not always situated within contemporary linguistic theories. In the first part of the paper, I will interpret certain philosophers to be claiming that *good* is uniform across constructions with respect to its *arguments*. Not only does this interpretation help to situate these claims within linguistic theories, but it allows various types of uniformity thesis to be disentangled. I will distinguish strong and weak uniformity theses—based on whether they posit the exact same number and type of arguments for all of the above *good*-constructions, or merely some argument associated with all of them—and ones that concern arguments at a syntactic or solely semantic level.

In the second part of the paper, I will oppose all such uniformity theses. Not only is the motivation for them weak, but a promising strategy can be deployed against them. An illustration of one version of this strategy will draw on under-appreciated data about so-called *tough*-adjectives. I will claim that there is better motivation for alternative types of analyses of *good*, which I call ‘non-uniformity contextualism’ and ‘relativism’. By rejecting uniform arguments, these alternative analyses avoid commitment to syntactically contentious and cross-linguistically implausible views. The picture that emerges is one where the apparent diversity of *good*-constructions is reflected in their analysis.

This paper only targets uniformity theses about *good*-constructions. There is no in principle reason why apparently diverse constructions should not involve the same arguments at some level. Hence analogous claims about the uniformity of other constructions may well withstand linguistic scrutiny. Yet the paper will provide a way of clarifying such claims, and a strategy for opposing them, that may be adapted for other debates.

## Clarifying uniformity theses

### Arguments and adjuncts

Claims about a uniform form for *good* can be clearly evaluated only when situated within contemporary linguistic theories. Philosophers who advance these claims sometimes mention the *arguments* of *good*.^1^ However, a definition of this linguistic term is typically not provided. An initial task is to reach a linguistically informed but theory-neutral working definition of *arguments*.

The following intuition is regularly used to motivate a distinction between arguments and adjuncts: if one expression is required in a construction in order to make a second expression complete (or closer to being complete), then the first expression is an argument of the second. In the construction *Ylva sees Xavier*, the expression *Xavier* is required as part of completing the expression *sees*. In *Ylva sees Xavier clearly now*, neither *clearly* nor *now* is required to complete any other expression. Theorists often characterise arguments solely via this intuitive gloss with some examples, especially if the linguistic details are orthogonal to their discussion (e.g., Nebel, 2019, 67). Yet this intuition is not enough to uncover the nature of arguments and a means of identifying them. For instance, in the construction *Ylva ate liquorice*, is *liquorice* required as part of completing the expression *ate*? And if not, does this mean that it cannot be an argument?

Linguists aim to provide more detailed characterisations that capture this motivating intuition, although there is divergence regarding how to do so.^2^ A common approach begins with the idea of a mathematical *function*, which applies to a specific number of *arguments* in order to output a value. It is natural to think of functions as incomplete in themselves, whereas applying them to the appropriate number of arguments yields a complete non-function. Linguistic theories make use of *syntactic functions* as part of their analysis of grammatical structure, where these apply to and output syntactic categories (or other forms of syntactic representation). Linguistic theories also make use of *semantic functions* as part of their analysis of meaning, where these apply to and output denotations. Accordingly, we can distinguish *syntactic arguments* as the inputs to syntactic functions and *semantic arguments* as the inputs to semantic functions. Semantic arguments are sometimes categorised according to certain characteristic roles, such as agent, theme and experiencer (which respectively identify something that performs, undergoes or experiences a described action or event). For example, the syntactic function contributed by *sees* might apply to an object DP (determiner phrase) argument and output a constituent of category V$'$, such as *sees Xavier*. The semantic function contributed by *sees* might apply to a theme argument such as the individual Xavier and output the set of all individuals who see Xavier. Most theorists then describe an *expression* as an ‘argument’ of another expression when it supplies a *syntactic* argument, and as an ‘adjunct’ when it supplies a *syntactic function* (e.g., Ackema, 2015, 269).^3^ After all, the intuition that motivates the distinction appears to track the requirements for a construction to be syntactically complete. I will follow this usage, and will make it clear when discussion instead centres on an expression’s supplying a *semantic* argument.

A straightforward picture would be one where each pronounced argument expression could be paired with a semantic argument that it denotes, and vice versa. Yet such a picture does not obtain. For a start, while argument expressions only ever supply semantic arguments and adjuncts typically supply semantic functions, there are potentially cases where adjuncts supply semantic arguments (Hole, 2015, 1302). More importantly, there might be cases of *implicit arguments*: semantic arguments supplied by a construction despite the apparent absence of an expression that denotes them. For instance, short passives, like *The spider was chased*, are naturally understood to involve an agent despite the apparent lack of a pronounced expression that denotes it (c.f. *The spider was chased by Xavier*). Similarly, psychological adjective constructions, like *The spider was frightening*, are naturally understood to involve an experiencer (c.f. *The spider was frightening to Xavier*). Implicit arguments are analysed in one of two broad ways.

First, a corresponding syntactic argument might be provided by an element that is present at some level of syntactic structure despite not being pronounced (Landau, 2010; Manzini, 1992; Collins, 2017). Second, there might be a semantic argument that lacks a corresponding syntactic argument: a level of representation that is intermediate between syntactic structure and any assignment of denotations—such as a theta grid, argument structure, discourse representation structure, or logical form—might include syntactically unrealised positions or variables that are interpreted as semantic arguments (Williams, 1987; Bruening, 2021; Rizzi, 1986; Condoravdi & Gawron, 1996),^4^ Those who analyse at least some implicit arguments in the second way allow there to be fewer syntactic arguments than semantic ones. Perhaps *The spider was frightening to Xavier* includes an optional syntactic argument contributed by *to Xavier*, whereas *The spider was frightening* lacks this type of syntactic argument but is nevertheless associated with an implicit agent argument. Theorists who analyse all implicit arguments in the first way maintain that an expression’s argument is always present, but it might sometimes be a ‘silent’ element that is not pronounced.

Both types of analysis of implicit arguments introduce theoretical complications. On one hand, while the potential for unpronounced elements is widely accepted within generative syntax, theorists aspire to avoid positing gratuitous ones. On the other hand, the second type of analysis remains controversial because syntactic relations sometimes seem to emerge between correlates of implicit arguments and other constituents, and the idea that syntactically unrealised items participate in these relations is deemed ‘conceptually problematic’ (Bhatt & Pancheva, 2006, 581). More generally, approaches that derive semantic interpretations directly from the output of syntax (e.g., Heim & Kratzer, 1998) might reject in principle the idea of semantic elements without syntactic correlates.

Given the complications posed by implicit arguments, why not simply deny their existence and classify all optionally pronounced expressions as adjuncts? Then, the straightforward picture where semantic arguments correlate perfectly with pronounced argument expressions might be sustainable after all. An answer emerges by reflecting on the typical methods that are used to indicate the presence of an argument. I will mention three methods for argument expressions, and then two for implicit arguments.

One method observes that pronunciation of the target expression is required in order to yield a syntactically complete construction. Of course, not all candidates for arguments are obligatorily pronounced: *The spider was frightening* is not obviously incomplete. Hence a second method draws on theoretical considerations. For example, a hypothesis of certain theories in generative syntax might entail that *by Xavier* is an argument (as opposed to an adjunct) in *The spider was chased by Xavier* (see Collins, 2005). A third method draws on theory-neutral tests, such as the following: only adjuncts can ‘float’ within their clauses to maximal projections, a verb’s argument must undergo ellipsis along with the elided verb while an adjunct need not, and an expression and its argument generally cannot be separated by an intervening adjunct (Collins, 2013). For instance, in a psychological adjective construction, *to Xavier* can neither ‘float’ (?*The spider to Xavier was frightening*) nor occur after an intervening adjunct (?*The spider was frightening on Sunday to Xavier*).

When motivating an implicit semantic argument, one method provides evidence of certain syntactic relations that have been claimed to require a syntactically realised argument (see Landau, 2010, 368–370). However, not all implicit arguments will be of the right type to allow the relevant syntactic relations, and this strategy is inapplicable to implicit arguments that are deemed to be syntactically unrealised. A second method shows that the putative implicit argument behaves like one of two categories recognised in the literature, namely *definite* (a specific value for the implicit argument must be recoverable from the context) or *existential* (no specific value needs to be identified; Condoravdi & Gawron, 1996).

The above methods often provide evidence in favour of classifying something as an argument expression or an implicit argument. The fact that these methods might support such a classification for certain optionally pronounced expressions is why theorists refrain from classifying all optionally pronounced expressions as adjuncts. Hence when an expression is optionally pronounced in a construction—such as *to eat* in *Liquorice is good for children to eat*—this does not preclude analysing it as an argument expression and as denoting a potentially implicit argument. The construction *Liquorice is good* could theoretically involve the same semantic arguments as *Liquorice is good for children to eat*, with those arguments merely lacking a pronounced correlate.

In sum, the most straightforward linguistic picture would be one with exact correlation between *syntactic arguments* (the input to functions from syntactic categories to syntactic categories), *semantic arguments* (the input to functions from denotations to denotations) and pronounced argument expressions that supply the former. Then, syntactic and semantic arguments could be identified based on the expressions that must be pronounced in order to make another expression complete. The potential for *implicit arguments* (semantic arguments supplied by a construction without an obvious expression that denotes them) makes such a picture unattainable. Accounts of implicit arguments might hold that there is an unpronounced but syntactically real expression that provides the corresponding syntactic argument, or that there is some syntactically unrealised element at an intermediate level of representation. Either way, which expressions are pronounced is not an infallible guide to which syntactic and semantic arguments are present. The methods that are typically used to identify argument expressions and implicit arguments are thus indirect and defeasible. Still, it is the potential for implicit arguments that opens the door to uniformity theses.

### Different uniformity theses

I interpret philosophers’ claims about uniform form to concern the arguments associated with *good*. As mentioned, philosophers who attribute a uniform form to constructions with *good* or other words sometimes mention uniformity of *arguments*. Combining this interpretation with the above characterisation of arguments helps to situate claims about uniformity within contemporary theories of syntax and semantics. It also allows various types of uniformity thesis to be distinguished. The first distinction concerns strength.

*Strong uniformity:* In (1a)–(1m), *good* contributes an element that applies to the same number and type of arguments.

*Weak uniformity:* There is at least one type of argument to which an element contributed by *good* applies in all of (1a)–(1m).

Note that advocates of strong uniformity are not committed to a claim about all *good*-constructions whatsoever, which might go beyond what any philosopher would endorse. Rather, I interpret uniformity theses to centre on a certain array of *good*-constructions, which for current purposes I take to be (1a)–(1m).

The diversity of the constructions with respect to their pronounced form means that advocates of uniformity views will often have to posit implicit arguments. Advocates of strong and weak uniformity might be further divided according to the nature of the arguments they posit for constructions that lack pronounced correlates.

*Syntactic uniformity:* A uniformity thesis holds with respect to syntactic arguments (and also with respect to the corresponding semantic arguments).

*Semantic uniformity:* A uniformity thesis holds solely with respect to (syntactically unrealised) semantic arguments.

For example, consider the idea that the denotation of *good* applies to a noun’s denotation as a semantic argument not only for sentences like *That is a good knife*, but also for ones such as *It is good to have pets*. A syntactic uniformity view would posit an unpronounced but syntactically real noun, present in the appropriate position in the syntactic representation of the latter sentence. A semantic uniformity view would deny that there was any such silent element but might hold that a logical form, intermediate between syntactic structure and assignment of denotations, contains a variable that gets a noun denotation as its value. The distinction between these views might seem subtle. Yet there are linguistically important differences between them. For instance, advocates of syntactic uniformity theses predict the potential for detectable syntactic evidence of the implicit arguments of *good*. Advocates of semantic uniformity theses must characterise the nature of the intermediate level of representation at which correlates of the arguments occur.

In short, when uniformity theses about *good* are situated within contemporary linguistic theories, they incur syntactic and semantic commitments. These commitments make it easier to evaluate them. The remainder of the paper undertakes this evaluation.

## Why endorse uniformity?

### Motivations

What would motivate a uniformity thesis about *good*-constructions? To my knowledge, no existing theorist has pursued the methods that provide the strongest motivation for implicit arguments (see §2.1): giving evidence of syntactic relations that indicate a syntactically realised implicit argument, or showing that the hypothesised implicit argument exhibits the behaviour expected of either a definite or existential one. Perhaps these methods have not been applied to *good*-constructions because they would not succeed. Advocates of uniformity theses would then need to pursue alternative strategies.

One popular way of motivating a uniformity thesis is to draw attention to the apparent *context-dependence of occurrences of good*. An occurrence of *This knife is good* might seem true relative to one context and false (with respect to the same knife) relative to another context. Many theorists have concluded that occurrences of *good* involve an obligatory element that contributes a perspective, role, end, experiencer or judge (e.g., Silk, 2021; Szabó, 2001; Bylinina, 2017). Given the obligatory nature of this element, it might be inferred that it would be an argument of *good*. Context-dependence would emerge either because the element itself always receives a contextually supplied value, or because the frequent absence of a pronounced correlate of that element results in implicit arguments that have the contextually recovered values characteristic of definite implicit arguments. This reasoning might be taken to motivate a weak uniformity thesis about at least semantic arguments. The flaw in the reasoning is that a covert element linked to *good* might be included as part of the lexical entry without constituting an argument. This possibility is discussed further in §5.

Another attested motivation is *intuitions concerning clarified semantic interpretation*. Finlay claims that ‘If the proposition expressed by a use of ‘good’ involves some implicit argument, then it will be clarified and explicated, rather than modified, by making that argument explicit’ (2014, 23). In other words, if an occurrence of *Liquorice is good* is associated with an implicit argument, then a variant of the construction with a pronounced expression that denotes the implicit argument will result in a truth-conditionally equivalent semantic interpretation (i.e., uttering either variant at a context will result in the same truth-conditions). He further claims that the additional phrases in (1a)–(1m) ‘complete different parts of the logical form of ‘good” (2014, 26). Hence an occurrence of *Liquorice is good* might be deemed to have an equivalent interpretation to *Liquorice is good for children to eat as a bribe*. Finlay’s further claim could be used to support a strong uniformity thesis, where the same multitude of arguments are present in (1a)–(1m) at either a syntactic or purely semantic level.

The trouble with this approach is that there are cases of merely apparent implicit arguments, where the construction provides no semantic argument yet interpreters pragmatically infer that the described state of affairs includes a participant that plays a certain type of role. This inference might be so natural that assessors mistakenly judge a variant of the construction with a pronounced expression that denotes the inferred participant to clarify the semantic interpretation of the original. For example, suppose that *Ylva ate* is a complete construction and involves no implicit arguments. Interpreters might nevertheless infer that any eating event will involve a theme consisting of something that is eaten and a location where the event takes place. This is a natural inference based on world knowledge. This might lead the interpreter to judge constructions like *Ylva ate something somewhere* or *Ylva ate liquorice in the kitchen* to clarify the semantic interpretation of the occurrence of the original. Yet in the envisaged case, the latter variants yield different semantic interpretations with different truth-conditions. Furthermore, even in cases where a variant genuinely clarifies the semantic interpretation of a construction that includes a covert element, it need not follow that the covert element is an *argument* of any expression in the construction. The covert element might instead supply a syntactic or semantic function, or it might be specified as part of some expression’s lexical entry (see §5). Introspective judgements of clarified semantic interpretation are not a reliable method of detecting genuine semantic arguments.

A further attested motivation is *parsimony*. Finlay states that ‘The default hypothesis should be that ‘good’ has a single, unified semantics’ (2014, 19). The suggestion is that we should attempt to develop a semantic analysis of *good* where all occurrences are associated with a single entry in the lexicon, are represented in the same way at any intermediate level of semantic representation, and are assigned the same denotation taking the same number and type of semantic arguments. We should pursue an alternative analysis only if it becomes clear that such a project faces insurmountable challenges. This would motivate a strong uniformity thesis, at least about purely semantic arguments.

There might be grounds to reject the default hypothesis about absolutely all occurrences of *good*. Wolfsdorf (2019) argues that *good* is three-ways ambiguous between evaluative *good* (which occurs in (1a)–(1l)), quantitative *good* (e.g., *It’s a good distance to the shops*) and operational *good* (e.g., *The eggs are good, it’s the milk that’s off*). Mankowitz (2024) argues that there is a further disambiguation consisting of moral *good* (e.g., *She’s a good person*, along with the natural interpretation of (1m)). Yet even if there are multiple disambiguations, a unified semantic analysis could still be treated as the default hypothesis for each disambiguation of *good*. Of course, advocates of uniformity typically extend their thesis to at least the sentences in (1a)–(1m) (e.g., Geach, Finlay), and might deny that *good* is ambiguous. The point is just that a theorist who accepted ambiguity could still use parsimony to motivate a uniformity thesis for the ‘evaluative’ occurrences of *good* in (1a)–(1l).

A final motivation for uniformity is *metaphysical views about the primary bearers of value*. Shanklin (2011, 40) mentions this idea, although he treats it as a reason to reject the metaphysical view: ‘Any meta-ethical view according to which (e.g.) states of affairs are the primary bearers of goodness, entails a syntactic theory according to which ‘good’ is a predicate of only the one sort of syntactic object (e.g. complement clauses)’. That is, a theorist who holds that there is one type of primary bearer of value (states of affairs, individuals, etc.) might expect that the denotation of *good* will always apply to a semantic argument of the appropriate kind. While the metaphysical view does not plausibly *entail* a uniformity thesis—the possibility should not be excluded that English encodes a semantic meaning for *good* that fails to reflect the genuine bearers of value—it is reasonable to think that its advocates should expect or pursue one. Hence the view that there is one type of primary bearer of value motivates at least a weak semantic uniformity thesis.

In sum, existing advocates of uniformity theses do not deploy the pair of methods that provide the strongest motivation for implicit arguments. Alternative strategies have been pursued with varying success. On one hand, even if there is evidence of an obligatory context-dependent element, it will turn out that such an element need not be an argument of *good*. Intuitions concerning clarified semantic interpretation do not provide a reliable means for detecting genuine semantic arguments (as opposed to pragmatically inferred participants or covert elements that do not count as arguments). On the other hand, parsimony motivates a strong uniformity thesis about at least semantic arguments, as the default hypothesis for at least the ‘evaluative’ occurrences of *good* in sentences like (1a)–(1l). Furthermore, the view that there is one type of primary value-bearer motivates at least a weak semantic uniformity thesis. Later parts of the paper will claim that even the most promising motivations do not justify upholding uniformity theses.

### Advocates of uniformity

Even after clarifying different versions of uniformity theses and their motivations, it might be asked: how would one develop a uniformity thesis in detail? The current section completes the illustration of the paper’s target views by describing two of the most well-developed uniformity theses.

#### Geach

Geach (1956) is generally interpreted as suggesting that the denotation of *good* requires a semantic argument consisting of the denotation of a noun. He begins by drawing a ‘logical distinction’: in a construction of the form *an A B*, “A’ is a (logically) predicative adjective if the predication ‘is an A B’ splits up logically into a pair of predications ‘is a B ’ and ‘is A’; otherwise I shall say that ‘A’ is a (logically) attributive adjective’ (1956, 33). This distinction is presumably drawn at the level of a logical form that represents semantic meaning. For instance, the adjective might be deemed logically predicative in *is a sharp knife* and attributive in *is a former knife*: a logical form that represents the semantic meaning might reasonably be split up into components that can be paraphrased as *is a knife* and *is sharp* in the former case, but not *is a knife* and *is former* in the latter case. Geach implies that logically attributive occurrences of adjectives should be analysed as supplying functions that require an argument supplied by a noun in order to denote a property of individuals. Geach then claims that *good* is always logically attributive: ‘Even when ‘good’ or ‘bad’ stands by itself as a predicate, and is thus grammatically predicative, some substantive has to be understood’ (34). Some Geach-style logical forms are paraphrased below, with potential values for *x* given in parentheses^5^:

-  Liquorice is good to eat.
  * Liquorice is a good x to eat. (x = food.)*
-  That is a good knife.
  * That is a good knife & is a knife.*

Geach commits himself to a weak uniformity thesis: while one argument is claimed to be present in each of (1a)–(1m), the possibility is left open that some of those sentences involve additional argument expressions or implicit arguments. His thesis appears to concern purely semantic arguments: *good* is always ‘logically attributive’ but might still be ‘grammatically predicative’. In cases where *good* is ‘grammatically predicative’, presumably a syntactically unrealised element of the logical form would supply an implicit argument.

#### Finlay

Finlay (2014) claims that all occurrences of *good* denote ‘relational properties’ that take three propositions as semantic arguments. The first argument specifies an end, the second specifies conditions that potentially increase the probability of that end, and the third provides a ‘background’ that constrains the relevant alternative worlds in which those conditions obtain or fail to obtain. He thus attributes to (1a)–(1m) logical forms that can be paraphrased via the schema: *It is good *[*for e*] [*if p*],* (given) *[*b*]. Finlay’s logical forms are paraphrased below, and the same types of expressions (infinitival phrases, nouns, etc.) in other *good*-constructions will contribute to logical forms analogously. Variables’ values are provided by the context, and the values of $s_1$ and *v* combine to form a proposition that specifies an end (that one has pleasure, that Ylva’s poker opponents are defeated, etc.).^6^

- Liquorice is good to eat.
  *It is good for *$s_1$’*s* *v-ing, for *$s_2$* to eat liquorice *(*given b*).
-  Ylva is good at playing poker.
  * It is good for *$s_1$’*s* *v-ing, for Ylva to be the one engaged at playing poker *(*given b*).
-  That is a good knife.
  * That is a knife such that it is good for *$s_1$’*s* *v-ing, for *$s_2$* to *$v_2$* it *(*given b*).

Finlay endorses a strong uniformity thesis: the denotation of *good* applies to exactly three proposition arguments in (1a)–(1m). However, it is difficult to tell whether the thesis concerns syntactic arguments. On one hand, Finlay (2020, 141) states that his analysis ‘seeks an ambitiously unifying syntax and semantics underlying all these different forms’. On the other hand, Finlay’s conception of a logical form appears to be more closely aligned with a representation of semantic meaning. Moreover, Finlay frequently motivates the presence of elements in the logical form by using introspective judgements of clarified semantic interpretation (see §3.1). Such judgements are relevant only to the project of motivating implicit arguments that are represented at an intermediate level, leaving open the issue of syntactic realisation. Hence this approach is most charitably interpreted as an attempt at motivating semantic uniformity theses.

#### Other uniformity theses

Several other uniformity theses have been proposed. Lassiter (2015, 147) and Morzycki (2016, 41–42) sketch a weak syntactic uniformity thesis centring on an *as*-phrase argument, although they do not commit themselves to it. Shanklin (2011) develops a weak semantic uniformity thesis involving a semantic argument consisting of a value scale. Semantic representations suggested by these two views are respectively paraphrased as below:

-  Liquorice is good to eat.
  * Liquorice is good as an x to eat. (x = food.)*
  * Liquorice has a degree on scale X in excess of a contextually salient threshold. (X = a   scale measuring gustatory value.)*

There are some other recent views that might appear to count as uniformity theses, but fail to meet the current criteria to count as such. Silk (2021) develops an analysis of a class of ‘evaluational adjectives’ that includes *good*. His official account takes such adjectives to contribute variables to semantic representations, where these variables are interpreted as ‘measure functions’ (functions from individuals to degrees on a scale). Szabó (2001) analyses *good* as an ‘incomplete one-place predicate’, insofar as its semantic representation includes a variable that receives a contextually assigned value consisting of a ‘role’ in which something can be good. Neither of these views would count as uniformity theses: the adjective’s semantic representation would end up as *good*$_v$, so the context-dependent element would be part of the lexical entry of the adjective itself, rather than syntactically combining with it as either an argument or adjunct.^7^ This type of non-uniformity view will be returned to in §5.

## Against uniformity

The previous section illustrates the diversity of existing uniformity views about *good*-constructions. Geach’s weak semantic uniformity thesis posits a semantic argument consisting of the denotation of a noun. Finlay’s strong uniformity thesis concerns at least semantic arguments, and takes *good* to denote a property that requires three propositions as arguments. Other theorists, such as Lassiter and Shanklin, develop further types of uniformity theses. The typical methods that linguists deploy to indicate the presence of arguments have not been used to motivate these uniformity theses. The alternative approaches that have been pursued show varying degrees of success. The strongest motivations come from aspirations of parsimony and metaphysical views about value-bearers.

Given this diversity amongst uniformity views, how can they be opposed? A promising strategy takes the following form. The first step is to identify an instance of a pronounced argument expression of *good* in (1a)–(1m). The second step is to identify a sentence amongst (1a)–(1m) where there appears to be neither that pronounced argument nor a corresponding implicit argument. Both steps may draw on the typical methods for motivating the presence of arguments discussed in §2.1: the classification of a pronounced expression as an argument may be supported by its essential role in yielding a complete construction, theoretical considerations and theory-neutral tests; and the presence of an implicit argument may be supported by certain syntactic relations, or behaviour characteristic of one of the two types of implicit argument.

Successfully carrying out this two-step strategy for one case opposes strong uniformity. Successfully carrying it out for all plausible candidates for arguments present in (1a)–(1m) additionally opposes weak uniformity. In §4.1, I will pursue a version of the strategy that draws on observations about *tough*-adjectives. This will provide evidence that (1j)–(1k) involve an infinitival or prepositional phrase argument that is absent from some other constructions. In §4.2, further applications of the strategy will be illustrated.

### *Good* as a *tough*-adjective

#### *Tough*-adjectives and their arguments

*Tough*-adjectives are a target of long-standing debate within generative syntax. They can occur in *tough-subject constructions* (e.g., (2a)) in which the subject DP is linked to a non-subject gap in an infinitival clause.^8^ Their defining feature is the potential to also occur in syntactically acceptable *expletive constructions* (e.g., (2b)) that appear to have truth-conditionally equivalent semantic interpretations to *tough*-subject variants. This feature distinguishes them from a class of *pretty*-adjectives, which occur in constructions that are superficially similar to *tough*-subject constructions (e.g., (2c)) but lack acceptable expletive variants (e.g., (2d)).

(2) a. Xavier is (tough / easy / important / impossible) to please $\_$.
  b. It is (tough / easy / important / impossible) to please Xavier.
  c. Xavier is (pretty / delicious / graceful) to look at $\_$.
  d. $*$ It is (pretty / delicious / graceful) to look at Xavier.

Another difference between *tough*-adjectives and *pretty*-adjectives is that the latter more naturally support entailments from a construction with an infinitival phrase to a construction without that phrase; indeed, *tough*-constructions typically sound unacceptable without an infinitival.

(3) a. Xavier is (pretty / delicious / graceful) to look at.
  b. $\Rightarrow$ Xavier is (pretty / delicious / graceful).
  c. Xavier is (tough / easy / important / impossible) to please.
  d. $\nRightarrow$ ? Xavier is (tough / easy / important / impossible).

*Tough*-adjectives continue to be a topic of debate due to difficulties in accounting for the connection between the expletive and *tough*-subject variants, and for certain puzzling features of the latter.^9^ For current purposes, the crucial point is that there are strong grounds to think that *tough*-constructions involve obligatory arguments. Virtually all analyses hold that, in either the expletive variant or in both constructions, there is an argument consisting of a prepositional phrase (Jones, 1983; Bylinina, 2014), an infinitival phrase (Lasnik & Fiengo, 1974; Keine & Poole, 2017; Hornstein, 2001; Rosenbaum, 1965; Chomsky, 1977) or both (Chung, 2001; Hicks, 2009; Pesetsky, 1987; Bresnan, 1971).^10^

The important role of infinitival *to*-phrases in *tough*-constructions should already be clear: an expletive variant is a grammatical clause only with such a phrase (e.g., $*$*It is (tough / easy / important / impossible)*). The same requirement typically holds for *tough*-subject variants when the adjective is interpreted with its *tough*-adjective sense (see (3d)). This provides initial motivation to classify the infinitival phrase as an argument, given that one method for motivating arguments is to show that an expression is required for a construction to be complete. The relevance of prepositional phrases (PPs) becomes clear once it is recognised that *tough*-constructions can always occur with a *for*-phrase that precedes the *to*-phrase. This *for*-phrase often seems to supply an agent who experiences the event (e.g., Ylva as the one who experiences the toughness of pleasing Xavier):

(4) a. Xavier is (tough / easy / important / impossible) for Ylva to please.
  b. It is (tough / easy / important / impossible) for Ylva to please Xavier.

Linguists have additional theoretical reasons for treating infinitival or prepositional phrases as arguments. For instance, those who claim that the *tough*-subject variant is derived by movement of the *tough*-subject from the object DP position of an infinitival clause (see fn.9) require *tough*-constructions to always include a pronounced or unpronounced infinitival. Linguists have also used theory-neutral tests to support argument status (Chung, 2001, 184–198; Bylinina, 2014, 23–27). For example, it has been claimed that, at least for the *tough*-subject variant, no adjunct may intervene between the adjective and the infinitival (e.g., ?*Xavier is tough on Sundays to please*).^11^ Similarly, it has been claimed that no adjunct may intervene between the adjective and an expression that is unambiguously a PP (e.g., ?*It is important on Sundays to Ylva to please Xavier*; see fn.11).

Linguists have taken *tough*-constructions to involve an implicit argument whenever the posited argument expression is unpronounced. As discussed above, the fact that *tough*-constructions typically require a pronounced infinitival phrase has been claimed to support its status as an argument. Still, there are cases where a *tough*-subject variant appears acceptable without a pronounced infinitival:

(5) The exam is (tough / easy / important / impossible).

It has been pointed out that such cases are restricted to ones where the denotation of an omitted infinitival is contextually recoverable, either due to a sufficiently rich discourse context or due to characteristics that are typically associated with the denotation of the *tough*-subject (Hicks, 2009, 538). For instance, typical characteristics of exams make it natural to understand (5) as equivalent to (6a). In a context where speakers instead intend to convey the information expressed by an occurrence of (6b), it is clear that (5) would not be an appropriate substitute. Moreover, in cases where the denotation of an infinitival fails to be contextually recoverable—such as in (3d) and (6c)—an omitted infinitival is unacceptable (c.f., (6d)).

(6) a. The exam is (tough / easy / important / impossible) to pass.
  b. The exam is (tough / easy / important / impossible) to (fail / talk about).
  c. ? That the exam is a fix is (tough / easy / important / impossible).
  d. That the exam is a fix is (tough / easy / important / impossible) to deny.

Similar constraints on contextual recoverability are characteristic of definite implicit arguments. This might be taken to support the view that all *tough*-constructions involve a semantic argument denoted by an infinitival, whether or not an infinitival is pronounced. One attempt at motivating the view that *tough*-constructions involve an implicit experiencer argument draws on potential syntactic evidence that a PP is syntactically realised even when unpronounced (see Berman & Szamosi, 1972, 322).

It cannot be ruled out that conflicting considerations will eventually convince theorists that both infinitival and prepositional phrases are adjuncts.^12^ Still, the majority of current theorists have concluded that *tough*-adjectives require at least one type of argument, and that *tough*-constructions where no such argument is pronounced involve a corresponding implicit argument.

#### Implications for *good*

What does the investigation of *tough*-adjectives have to do with *good*? I will argue that *good* occurs as a *tough*-adjective. Hence there are strong grounds for thinking that such occurrences of *good* involve an infinitival or prepositional phrase argument. Yet I will also argue that *good* sometimes occurs as a *pretty*-adjective, and that it is far from obvious that *pretty*-adjectives require the same arguments as *tough*-adjectives. This will constitute one application of the two-step strategy for opposing uniformity theses.

Several theorists have classified *good* as a *tough*-adjective (Berman & Szamosi, 1972, fn. 20; Chung & Gamon, 1996, 61–63; Shanklin, 2011, 73–74; Bylinina, 2014, 46).^13^ Such a classification is motivated by sentences like the following, where syntactically acceptable expletive constructions appear to have equivalent semantic interpretations to *tough*-subject variants:

(7) a. Xavier is good to please.
  b. It is good to please Xavier.
  c. Pets are good (for Xavier) to have.
  d. It is good (for Xavier) to have pets.

Moreover, *tough*-subject variants do not naturally support entailments to a construction without an infinitival phrase, and such a construction sounds somewhat unnatural out of context:

(8) a. The exam is good to take.
  b. $\nRightarrow$ ? The exam is good.
  c. Cholesterol is good to avoid.
  d. $\nRightarrow$ ? Cholesterol is good.

The potential for a *tough*-subject construction with *good* to occur without a pronounced infinitival phrase obeys the contextual recoverability constraints shown by *tough*-adjectives, in the manner that supported the presence of an implicit argument. For example, (8b) might be acceptable relative to a context where it is preceded by an utterance of *I’m deciding what the best assessments are to take*.

In short, there are constructions where *good* behaves exactly like a *tough*-adjective. Moreover, the apparent semantic equivalence of (7c) and (7d) strongly suggests that (1j) and (1k) are the expletive variants of *tough*-adjective occurrences of *good*. This completes the first step of the strategy for opposing uniformity theses. That is, evidence that *good* occurs as a *tough*-adjective in (1j) and (1k) entails that *good* is likely to take an infinitival or prepositional phrase argument in those sentences, given that virtually all analyses of *tough*-adjectives posit at least one of those arguments. The next step is to make the case that there are sentences amongst (1a)–(1m) where *good* lacks these arguments.

There are grounds to deny that *good* always occurs as a *tough*-adjective.^14^ First, not all *good*-constructions have acceptable expletive variants:

(9) a. Xavier is good with children.
  b. $*$ It is good with children Xavier.
  c. Xavier is good to Ylva.
  d. $*$ It is good to Ylva Xavier.

(9a) and (9c) depart from the surface form of *tough*-subject constructions in a crucial way: neither includes an infinitival phrase with a non-subject gap. Attempts at replacing the clause-initial DP with an expletive and moving it to the end of the construction are clearly ungrammatical.

It might be countered that there is an implicit argument denoted by an infinitival phrase, and a corresponding infinitival simply needs to be pronounced in order to make an expletive variant grammatical. For instance, it might be thought that (9a) has an equivalent interpretation to *Xavier is good to (leave / trust) with children*, and that an expletive variant of that sentence would also be an expletive variant of the original version. If *tough*-adjectives take a PP argument, then it might additionally be thought that the expletive variant of (9a) includes such a PP (e.g., *It is good for parents to (leave / trust) Xavier with children*). The main problem with this reasoning is that, as discussed above, *tough*-constructions typically sound unacceptable without an infinitival. Indeed, variants of (9a) with paradigm *tough*-adjectives are unacceptable (?*Xavier is (easy / important) with children*). It would need to be explained why *good* is an exception. Moreover, intuitions concerning clarified semantic interpretation are not a reliable method for detecting implicit arguments (see §3.1). Finally, there are *good*-constructions like (9c) where it is very difficult to come up with a candidate for a truth-conditionally equivalent variant with a pronounced infinitival or PP: it seems that no such expression can acceptably occur between *good* and *to Ylva*, or after the latter expression. The absence of acceptable expletive variants for some occurrences of *good*, in addition to the implausibility of implicit arguments that correspond to the ones selected by *tough*-adjectives, would provide evidence that *good* does not always occur as a *tough*-adjective.

Further evidence comes from the fact that, even when expletive variants are syntactically acceptable, truth-conditional equivalence is often questionable.

(10) a. Liquorice is good to eat.
  b. It is good to eat liquorice.
  c. This knife is good to (cut chives / stab people) with.
  d. It is good to (cut chives / stab people) with this knife.
  e. Pleasure is good for Xavier to experience.
  f. It is good for Xavier to experience pleasure.

(10b), (10d) and (10f) are naturally interpreted as suggesting that there is some moral or fairly general sense in which a described state of affairs has value, a suggestion that does not emerge for the variants. This difference is highlighted by considering (10e) and (10f) relative to a scenario described by Finlay (2014, 27–28): if Xavier has perpetrated horrific crimes, then it might be natural to judge an occurrence of (10e) to be true and an occurrence of (10f) to be false.

It is worth noting that there can be differences between expletive constructions and *tough*-subject variants, such as the widely reported impression that the *tough-*subject variant implies that the denotation of the subject DP is responsible for the toughness (etc.) of the described state of affairs. Plausible pragmatic explanations of such differences can be given (see Goh, 2000), and one might wonder if the apparent non-equivalence of (10a)–(10f) has the same source and solution. Yet there are grounds to expect a negative answer. Any differences between variants of (10a)–(10f) with paradigm *tough*-adjectives do not appear sufficient to raise doubts about *truth-conditional* equivalence: it is hard to imagine a context where occurrences of *Pleasure is (tough / easy / important / impossible) for Xavier to experience* and *It is (tough / easy / important / impossible) for Xavier to experience pleasure* would naturally be judged to differ in truth value. The impression of semantic differences between certain expletive and non-expletive variants appears to be more robust for *good* than for paradigm *tough*-adjectives.

A natural inference is that *good* has both *tough*-adjective and *pretty*-adjective disambiguations.^15^ For some non-expletive *good*-constructions, a *tough*-adjective disambiguation is barred, often for syntactic reasons (e.g., (9a)–(9d)). This corresponds with the absence of an acceptable expletive variant. For other non-expletive *good*-constructions, both disambiguations are in principle available, although one disambiguation might be more natural. An expletive variant will then be acceptable, but will force the *tough*-adjective disambiguation. The expletive variant will appear truth-conditionally non-equivalent to a non-expletive variant for which the *pretty*-adjective disambiguation is preferentially selected (e.g., in (10a)–(10f)), but might otherwise appear equivalent (e.g., in (7a)–(7d)).

This view has semantic and metaphysical implications, depending on one’s analyses of *tough*-adjectives and *pretty*-adjectives. Some theorists suggest that a *tough*-adjective’s denotation applies to an event, proposition or state of affairs argument denoted by an infinitival phrase, whereas a *pretty*-adjective’s denotation applies to an individual or generalized quantifier argument denoted by the clause-initial DP (Keine & Poole, 2017, 315–316; Gluckman, 2021, 465–466). *Pretty*-adjective constructions would then involve a semantic relation between the subject DP and the adjective, which might explain their natural entailments. Their infinitival clause might be an optional elaborative adjunct (Chung & Gamon, 1996, 62). It would follow that some occurrences of *good* describe an event, proposition or state of affairs, whereas others describe an individual or generalized quantifier. Advocates of metaphysical views where there is one type of primary bearer of value would need to reconcile their views with semantic accounts that involve at least two types.

This completes the second step of the strategy for opposing uniformity. For note that (1b) and (1e) were included amongst examples without acceptable expletive variants, and (1a) was included as an example where truth-conditional equivalence is questionable. The reasoning in the current section therefore indicates that some sentences amongst (1a)–(1m) lack the arguments selected by *tough*-adjectives. This conclusion follows particularly clearly if one accepts that *good* has disambiguations as a *tough*-adjective and as a *pretty*-adjective. First, there would be no reason to expect that any arguments required by a *tough*-adjective would be present—even as implicit arguments—for occurrences of a non-*tough*-adjective. Second, analyses of *pretty*-adjectives often deny that an infinitival or PP is an argument of such adjectives. A similar conclusion can be reached even if the ambiguity view is rejected: as argued above, it is independently implausible to claim that (1b) and (1e) involve implicit arguments corresponding to the arguments selected by *tough*-adjectives.

The reasoning in the current section targets both syntactic and semantic uniformity theses. The claim is not merely that some sentences amongst (1b)–(1e) lack syntactic elements corresponding to *tough*-adjectives’ arguments, but that they also do not involve syntactically unrealised implicit arguments. The reasoning also holds the potential to target specific uniformity theses and analyses of *good*. For example, analyses where *good* is always a *tough*-adjective or always has an infinitival phrase argument would be undermined by the current section.

### Further applications of the strategy

The strategy for opposing uniformity theses can be applied to other constructions and arguments. If a strong uniformity thesis is deemed to be successfully opposed by a detailed application of both steps of the strategy—such as the one targeting *tough*-constructions in §4.1—then particular weak uniformity theses may be opposed by just applying the second step. The reason is that identifying an argument present in one construction and absent from another is required to rule out strong uniformity theses that posit exactly zero arguments. However, uniformity theses that posit a particular type of argument are challenged by evidence that it is absent from some constructions, whether or not it is present for others. The current section sketches a further application of the strategy, with the aim of illustrating new ways of challenging weak uniformity theses in general.

To oppose Geach’s weak uniformity thesis (see §3.2.1), one must provide evidence against the presence of an implicit argument consisting of a noun’s denotation. It is not immediately obvious how to do this in English. Yet there are opportunities for a more straightforward application of the strategy if we look beyond English. Advocates of uniformity theses are likely to want to endorse cross-linguistic theses.

*Cross-linguistic uniformity:* A uniformity thesis holds with respect to translations of (1a)–(1m) in all languages.

One reason is that at least some of their motivations—namely, considerations of parsimony and metaphysical views about value bearers—would motivate cross-linguistic uniformity theses, not just uniformity theses about English. Another reason is that any broader semantic, metaphysical or meta-ethical implications of the linguistic analysis of *good* would be expected to correlate with cross-linguistic generalisations, not the potentially contingent features of a specific language. The alternative to endorsing cross-linguistic uniformity theses would be to either treat any insights as language-specific, or to hold that the English semantics for *good* are somehow privileged in their accordance with parsimony, metaphysical accuracy, and revelation of broader insights.

On the face of it, cross-linguistic uniformity theses are implausible. It is widely accepted that argument structure varies cross-linguistically (Comrie, 1993, 911). For example, the denotation of the English word *envy* can take three semantic arguments (e.g., in *Ylva envies Xavier his liquorice*) whereas the denotation of the Russian translation can only take two. Advocates of uniformity theses might try to claim that *good* and its translations are connected to some core human concept in a way that leads to stability of the number and type of semantic arguments across languages, or at least within each language. Whether or not cross-linguistic uniformity theses can be plausibly defended, they open up the potential for new applications of the anti-uniformity strategy.

As part of a broader debate about adjectives’ semantic type, it has been noted that Russian adjectives have a morphologically distinct ‘long form’ and ‘short form’ (Morzycki, 2016, 30–34). For example, *The student is (good / intelligent)* can be translated as either *Studentka* (*khoroshaya*$_{\textsc {long}}$ / *umnaya*$_{\textsc {long}}$) or as *Studentka *(*khorosha*$_{\textsc {short}}$ / *umna*$_{\textsc {short}}$). The two forms show syntactic and semantic differences. A prominent analysis developed by Siegel (1976) holds that long form adjectives in predicative position occur with implicit noun denotation arguments, but that short form adjectives in predicative position do not. This would oppose the weak uniformity thesis under current consideration, as a thesis about Russian or about all languages. While the correct analysis of the data remains a topic of ongoing debate,^16^ an anti-uniformity strategy based on Russian adjectives is an intriguing avenue for further exploration.

This subsection raises a further dilemma for advocates of uniformity theses. Cross-linguistic uniformity theses are prima facie implausible. They also allow new versions of the strategy for opposing uniformity, such as one targeting noun arguments. Yet advocates of uniformity theses are likely to want to endorse cross-linguistic theses, if their theorising is to yield insights that extend beyond contingent features of English.

## Implications

If uniformity theses are rejected, then what happens with respect to the motivations for them? For instance, the apparent context-dependence of occurrences of *good* seems undeniable. The current section describes alternative analyses of *good* that are arguably better motivated than those that endorse uniformity theses.

As hinted at various points, context-dependence need not be traced to a context-dependent *argument*. For example, indexicals like *now* and *she* are widely analysed as context-dependent: their interpretations will differ when used at six o’clock to talk about Ylva versus when used at midnight to talk about the spider. Yet this is not attributed to their having a denotation that combines with a contextually variant semantic argument. It is not the case that *she* is a syntactically incomplete expression in the construction *She sees Xavier*, in need of a context-dependent argument expression that supplies the individual. Rather, *now* and *she* are themselves analysed as context-dependent elements. Analogously, opponents of uniformity might recognise some context-dependent element associated with *good*, an element that we can represent as ‘*v*’. One option would implement the idea mentioned in §3.2.3, where *v* is introduced by the lexical entry and occupies the node for *good*. One might wonder how a context-dependent element could end up in the semantic representation of *good*, without being an argument of any other element. It is widely thought that an expression can be associated with a variable because its lexical entry specifies that a variable is the sole occupant or co-occupant of the node for that expression in a syntactic tree (Stanley & Szabó, 2000, 251).^17^ This variable would not count as an argument of the expression with which it is associated: it supplies neither a syntactic nor semantic argument for a function supplied by (say) *good*. It would not even count as an adjunct, because it does not supply a syntactic function either. (11) gives a rough representation of the resulting interpretation of *good* (abstracting away from details about constraints on the value of *v*), where $g_c$ is a contextually determined valuation function on variables:

(11) $$\llbracket $$ *good*$$_v$$ $$\rrbracket ^{g_c}$$ = $$\lambda x. g_c(v)(x)$$.

This approach is related to the contextualist analyses discussed in the literature on personal taste adjectives (Stojanovic, 2007; Stephenson, 2007), although that literature often does not distinguish context-dependent arguments from other syntactically realised elements. Hence let us call it *non-uniformity contextualism* about *good*.

A second option would treat *v* as a parameter of the interpretation function for *good*, which is neither syntactically realised nor present in the semantic representation. This would mean that *v* provides neither a syntactic nor semantic argument to an element contributed by *good*. A rough representation of the interpretation of *good*, which abstracts away from details about constraints on the value of *v* and the means by which it affects the denotation of *good* (represented as *G*), would be as follows.

(12) $\llbracket$ *good *$\rrbracket ^{g_c, v}$ = $\lambda x. G(x)$ relative to *v*.

This approach is a form of the relativist analyses in the literature on personal taste adjectives (Lasersohn, 2005; Bylinina, 2017). Accordingly, let us call it *relativism* about *good*.^18^

Importantly, both non-uniformity contextualism and relativism capture some of the motivations for uniformity theses. First, they make *good*-constructions context-dependent. Second, while intuitions concerning clarified semantic interpretation were deemed to be an unreliable means of detecting genuine semantic arguments, theorists who are convinced that a pair of *good*-constructions have truth-conditionally equivalent interpretations could explain this via non-uniformity contextualism or relativism. For example, if an occurrence of *Liquorice is good* is deemed to have an equivalent interpretation to *Liquorice is good for children to eat as a bribe*, then this might be attributed to a context-dependent element involved in the interpretation of both constructions such that the contextually assigned value is equivalent to the denotations contributed by *for children to eat as a bribe*. Third, non-uniformity contextualism and relativism respect a parsimony principle that recommends pursuing a unified semantics for *good* to the extent that this fits with syntactic evidence. Occurrences of *good* in (1a)–(1m) could be treated as unambiguous and traced to a single lexical entry. While *good* is not assigned the same denotation taking the same number of semantic arguments, this reflects the broader issue of distinct occurrences of adjectives and verbs’ appearing to provide semantic functions of different types—compare *Ylva is tall* with *Ylva is a tall woman*—and admits of a general solution. On the other hand, advocates of non-uniformity contextualism or relativism would also be free to argue that *good* is ambiguous and that only some disambiguations are associated with the context-dependent element; for example, it might be that moral occurrences of *good* lack the form of context-dependence under discussion, so occurrences in (1a)–(1l) involve a lexical entry that introduces the context-dependent element *v* whereas the occurrence in (1m) involves a separate lexical entry.

The clearest motivation to endorse uniformity theses over the alternatives would be the view that there is one type of primary bearer of value. After all, it is the *arguments* of *good* that are supposed to reveal primary value-bearers. Yet advocates of these metaphysical views will need to accommodate the evidence that *good* has *tough*-adjective and *pretty*-adjective occurrences with denotations that potentially apply to different types of things (see §4.1.2). An obvious way to accommodate this evidence would be to emphasise that the metaphysical views do not *entail* uniformity theses, given the potential for English to encode a meaning for *good* that fails to reflect the genuine bearers of value. Hence theorists who predict a single type of value-bearer are likely to ultimately reject a strategy of using metaphysical views to motivate uniformity theses. On the other hand, the motivations for uniformity theses that do withstand basic syntactic observations might be captured by non-uniformity contextualism and relativism. Therefore, there are no obvious motivations to endorse uniformity theses in particular.

The ways in which non-uniformity contextualism and relativism differ from uniformity views and from each other are quite subtle. One might then wonder about the broader significance of rejecting uniformity theses while endorsing one of the alternative analyses. Is the decision to classify a context-dependent element *v* as an argument or a non-argument merely notational, or trivial in some other sense? First, there might be different empirical predictions related to syntax and phonology. Like a syntactically realised argument, the element posited by non-uniformity contextualists could theoretically enter into syntactic relations. In contrast, the parameter posited by relativists could not enter into syntactic relations. On the other hand, a contrast emerges between uniformity theses and the alternative approaches with respect to the context-dependent element’s pronounceability: that is, whether an expression that compositionally provides the denotation or value of the element can be phonologically realised as part of the construction. It is plausible to think that *v* can never be pronounced in this way if it co-occupies a node for *good* or is a parameter of the interpretation function, and can be pronounced only as *good* if it is the sole occupant of a node. In contrast, the default expectation for a syntactic or semantic argument is that there will be some occurrences of the construction where it is pronounced as a fairly natural phrase (although there may of course be versions of the construction involving an implicit argument).^19^

Second, the rejection of the broader picture advocated by uniformity theorists has its own significance. This is a picture where the semantics, and possibly the syntax, of *good*-constructions show a basic consistency. It is also a picture where a syntactic or semantic argument is posited without deploying the typical methods that provide the strongest linguistic evidence for its presence. Finally, it is a picture that is committed to either the implausible prediction that uniformity theses will hold for translations of *good*-constructions cross-linguistically, or that any meta-ethical or metaphysical insights raised by a uniform linguistic analysis of *good* are linked exclusively to English.

Opponents of uniformity theses can reject this picture. According to the alternative analyses, the only consistent feature of the syntax and semantics of *good*-constructions is a context-dependent element linked to the interpretation of the adjective itself. Opponents of uniformity can avoid positing either extra nodes in the syntactic tree that lack syntactic evidence (see Collins, 2013), or syntactically unrealised semantic arguments (see §2.1). The rejection of a picture that allows syntactic or semantic arguments without adequate evidence underscores an important theoretical difference between uniformity and the alternatives. Finally, opponents of uniformity theses can grant the widely accepted view that there is cross-linguistic variation about which expressions are encoded as arguments (Comrie, 1993, 911). The rejection of a picture that posits either implausible cross-linguistic stability of argument status or lends excess weight to argument status in English is a further important theoretical difference between the pictures.

Hence a focus on defending uniformity theses over alternative approaches is misguided: many of the motivations for uniformity theses are captured by alternatives that avoid positing syntactic or semantic arguments, and there are grounds to think that argument status is encoded by individual languages with some arbitrariness. In this respect, the importance of defending a uniformity thesis over an alternative analysis of *good* is diminished. But there is another sense in which the choice between the approaches is highly significant, due to the empirical and theoretical differences discussed above.

## Conclusion

Despite the apparent diversity of *good*-constructions, a number of philosophers claim that there is something uniform about them. Such claims are, I contend, best situated within contemporary linguistic theories when understood to concern the arguments of *good*. The resulting *uniformity theses* then come in strong and weak versions—depending on whether there are claimed to be the same number and type of arguments for (1a)–(1m), or merely at least one type of argument for all of them—that centre on syntactic arguments or solely semantic ones. Theorists are also likely to endorse cross-linguistic versions of their uniformity theses, which hold with respect to translations of (1a)–(1m).

Uniformity theses have been motivated in several ways. These include evidence of context-dependence, intuitions of clarified semantic interpretation, the aim of a parsimonious analysis, and metaphysical views about value-bearers. I argued that the last two motivations are the most promising, but still do not successfully motivate uniformity theses. Indeed, analyses of *good* that reject uniformity theses can capture the first three motivations, by positing a context-dependent element as an occupant of the node for *good* (*non-uniformity contextualism*) or a parameter of the interpretation function (*relativism*).

A promising strategy for opposing uniformity theses begins by identifying an instance of a pronounced argument expression in (1a)–(1m), before identifying another one of the constructions that involves neither that argument expression nor a corresponding implicit argument. A version of this strategy was used to make the case that arguments corresponding to the infinitival or prepositional phrase arguments of *tough*-adjectives are present for (1j) and (1k) but absent for (1b) and (1e). If successful, this opposes not only all uniformity theses that posit the relevant arguments, but also strong uniformity theses in general. Further applications of the strategy can be used to target additional weak uniformity theses. One such application was mentioned, which raises doubts that a noun denotation argument is involved in all Russian translations of sentences like (1l) and (1m).

There are therefore grounds to accept alternative analyses of *good*, such as non-uniformity contextualism and relativism. These analyses capture most of the motivations for uniformity theses. Yet they better accord with syntactic, semantic and cross-linguistic generalisations. They also treat the apparent diversity of *good*-constructions as genuine.

## Footnotes

1 For example, Finlay (2014, 23) writes ‘If ‘good’ does have a unified meaning, then any kind of argument required for its logical completion in one case will be required for every meaningful use’.

2 The current paper emphasises arguments as the inputs to functions (following Hole, 2015). Other theory-neutral characterisations have focused on arguments as core participants in events (Ackema, 2015) or as required by a predicate (Comrie, 1993). All theory-neutral characterisations face difficulties in accommodating the distinction as formulated within every possible theory. For instance, a challenge for the current characterisation is that some theories include syntactic operations (e.g., Merge, in Chomsky, 1995) or semantic operations (e.g., Predicate Modification, in Heim & Kratzer, 1998) that combine pairs of elements without designating one as a function and the other as an argument. Moreover, theories often impose additional structural criteria, such as that one syntactic element counts as an argument of another only when the latter occupies the head position of the phrase and the former occupies complement or specifier position (see Jackendoff, 1977). There are even syntactic theories that make no distinction between arguments and adjuncts whatsoever (e.g., see Cinque, 2006). The current characterisation suffices to allow the ideas in the paper to be clearly expressed, but alternative characterisations could be substituted without affecting the paper’s conclusions.

3 I take an *expression* to be a lexical item or a constituent formed from lexical items. I use *element* in a broader way, to additionally cover occupants of syntactic tree nodes that would not count as expressions, along with parts of semantic representations. Note that some theorists reserve the term *argument* for what we here call *semantic arguments*, with *complement* or *specifier* referring to the corresponding expressions and their syntactic representations.

4 For example, Williams (1987) describes a version of this view involving *argument structures* which are representations of the number and type of semantic arguments required by an expression. The argument structure for *eat* in *Ylva*$_i$ *ate liquorice*$_j$ might be represented as: Williams associates implicit arguments with unfilled argument slots in argument structures. The argument structure for *eat* in *Ylva*$_i$ *ate* might then be thought to have an unfilled Theme slot.

5 There are subtleties involved in interpreting Geach that affect the logical forms, although these are not important for current purposes. For instance, Geach’s view might be that the logical form for a sentence like (1 h) should still include a variable (albeit with a value that is typically the denotation of the pronounced noun).

6 Finlay typically paraphrases the first proposition argument by using a possessive DP and a gerund form verb (e.g., *one’s having pleasure*), as opposed to a finite clause that obviously denotes a proposition. He typically paraphrases the second proposition argument by using the complementiser *for* rather than *if*.

7 Silk mentions an alternative implementation of his analysis, where evaluational adjectives contribute an element that places constraints on a variable argument that is interpreted as a measure function (2021, fn. 27). This would count as a weak uniformity thesis that centres on syntactic arguments. For Silk’s purposes, the choice between the two options is insignificant, given that they yield truth-conditionally equivalent denotations for occurrences of *good*.

8 Evidence for this link is that, for (2a), Xavier is the individual that potentially undergoes an event of being pleased. Moreover, the DP is pronounced in the gap position for the expletive variant.

9 The central puzzle is that the *tough*-subject appears to contribute a syntactic argument to an expression in the main clause along with a theme semantic argument to the verb of the infinitival clause, which risks violating certain theoretical principles (e.g., the theta criterion). Analyses fall under two main categories. *Movement* analyses hold that the *tough*-subject initially occupies the non-subject DP position of the infinitival verb for both expletive and *tough*-subject constructions, before moving to its surface position in the latter construction (e.g., Rosenbaum, 1965; Hartman, 2011). *Base-generation* analyses hold that the *tough*-subject initially occupies its pronounced position for both constructions, and that the gap position in the *tough*-subject variant is initially occupied by an unpronounced operator that ends up moving to a position where it can ‘share’ features with the *tough*-subject that constrain the type of semantic argument denoted (e.g., Chomsky, 1977; Gluckman, 2022).

10 Other than Hornstein, all of the cited theorists accept the same arguments for both variants. To my knowledge, the only theorists that explicitly classify both phrases as non-arguments are Rose (2018, 112–113) and Gluckman (2021, 2022).

11 One might then wonder how theorists who deny that there is a PP argument in *tough*-subject variants can avoid the incorrect prediction that sentences like (4a) are unacceptable due to an intervening *for*-phrase adjunct. It has long been recognised that the *for*-DP expression in *tough*-constructions is potentially ambiguous between a PP in the main clause (It is [$_{AP}$ easy [$_{PP}$ for Ylva$_i$] [ PRO$_i$ to babysit Xavier]], where *PRO* is an unpronounced pronoun, here interpreted as Ylva) versus a complementiser plus subject DP in the infinitival clause (It is [$_{AP}$ easy [$_{CP}$ for Ylva to babysit Xavier]]). Indeed, the expletive variant allows both *for*-phrases to occur simultaneously (*It is easy for the parents for Ylva to babysit Xavier*), although the *tough*-subject variant does not (?*Xavier is easy for the parents for Ylva to babysit*). A pressing question has been whether the solitary *for*-phrase permitted in the *tough*-subject variant is headed by prepositional *for* or complementiser *for*. Due to arguments developed by Hartman (2011), the latter option is now widely accepted (Keine & Poole, 2017). This is a major motivation for those who deny that there is a PP argument in *tough*-subject variants, and additionally avoids the incorrect prediction about the unacceptability of sentences like (4a): the *for*-phrase heads the infinitival argument of the *tough*-adjective, not an adjunct.

12 For instance, Gluckman (2022, pp. 260–264) queries whether the theory-neutral tests support argument status for the infinitival phrase in *tough*-constructions. Theorists sometimes respond by claiming that the relevant expressions are arguments with distinctive features that result in some adjunct-like behaviour (e.g., Chung,^2001^, 184–185; Bylinina, 2014, 23–27).

13 To my knowledge, Shanklin (2011) is the only philosopher to have considered the relevance of the literature on *tough*-adjectives to *good*. One limitation of Shanklin’s commentary is that the argument for classifying *good* as a *tough*-adjective rests on Pesetsky’s (1987) approach, which is now widely rejected (see Poole et al., 2023, 1–2). My own arguments remain neutral about the correct analysis of *tough*-constructions.

14 Similar observations have been made by Shanklin (2011, 75–77), Bresnan (1971, 266–268) and Lees (1960), with the latter two theorists positing a form of ambiguity. While Shanklin is emphatic that *good* is unambiguous, he claims that it takes a single theme-denoting argument in (1b), whereas it occurs as a *tough*-adjective that takes the same pair of arguments (PP and infinitival) in at least (1a), (1c), (1j) and (1k).

15 While theorists have mentioned other cases of adjectives with both disambiguations (e.g., *difficult*), many adjectives appear to belong exclusively to one of the classes (see Gluckman, 2021, 467). The following illustrates the grounds to think that some but not all *tough*-adjectives have *pretty*-adjective disambiguations: *My toddler is being (difficult / ? harmful / ? easy / ? simple*).

16 A recent analysis sketched by Martin and Bikina (2021) holds that the translation of *good* always occurs with a noun argument *except* when the long form occurs in predicative position. Despite the differences between this analysis and Siegel’s, the claim is still that a noun argument is present for some of the constructions and absent for others.

17 For example, if *good* heads a constituent of the adjective phrase category, then it is this head position that the variable would occupy. Hence even according to structural criteria, it does not occupy the right sort of position in a syntactic tree to count as an argument or an adjunct of *good*.

18 Note that both analyses are compatible with the metasemantic views that MacFarlane (2014) calls ‘contextualism’ and ‘relativism’. My adoption of the terms reflects an alternative common usage where ‘contextualism’ and ‘relativism’ correlate with whether a source of context-dependence is, respectively, part of the semantic representation or a parameter of the interpretation function (see Collins, 2013, 51–52; Bylinina, 2017, 296).

19 This expectation is often made tacitly (e.g., see Partee, 1989, 352), and is reasonable but defeasible: there do appear to be occasional examples of necessarily implicit, syntactically realised arguments Landau (2000, 159).
